# Sulphate of Quinine in Yellow Fever

**Published:** 1844-08

**Authors:** 


*Sulphate of Quinine in Yellow Fever.*—When I determine to administer sulphate of quinine, I order it in doses of two grains every hour for adults, until the system is saturated, which may be recognised by a species of intoxication and partial deafness, too well known to attend the exhibition of this medicine to be mistaken.

I believe that in a great number of cases where patients have been cured after having the quinine, the cure might have been obtained without the use of this remedy; but why neglect an agent which, were it only auxillary, always has the advantage when we employ it opportunely, to confirm a cure which, without it, would remain doubtful. I have sometimes attempted to dispense with its use, and in some few cases I have had reason to deplore its omission, for I had the misfortune to lose some patients in whom the remission was so complete, that the administration of quinine might certainly have effected a cure.

Heretofore the administration of the sulphate of quinine, as an anti-periodic during the remission, has not been called in question. But this is not the only mode of exhibiting this medicament; there is another of which I must speak. During the epidemic of 1841, I reflected whether instead of waiting until the remission was complete and permanent, to administer the sulphate of quinine, it would not be as natural and more efficacious to administer this remedy *de prime abord* during the short remission produced by a large syncopal bleeding.

Let me illustrate the position:—Suppose an individual in the onset of yellow fever is as stout as we could wish; a general bleeding is practised upon him, and this bleeding, when pushed to incipient syncope, induces all that series of phenomena which I have described above—phenomena which, in their *ensemble*, constitute a temporary but genuine remission. Well, can we avail ourselves of this momentary calm to administer the quinine in doses sufficiently large to create a profound perturbation, and break up that morbid chain on which the disease depends?

Such was the question which I propounded to myself in 1841—one which I resolved to test by clinical experience. After much hesitation, I determined to try the experiment, and the subject on whom it was made, was cured as by enchantment. Encouraged by this result, I repeated the experiment a second, then a third, and the fourth time, and always with the same success.—Dr. Beugnot in *New Orleans Med. Jour.*

